# Laryngeal Inflammatory Pseudotumour Secondary to* Mycobacterium kansasii*

**DOI:** 10.1155/2018/9356243

**Published:** 2018-07-09

**Authors:** Saif Al-Zahid, Tanwen Wright, Philip Reece

**Affiliations:** ^1^University Hospitals Bristol NHS Foundation Trust, UK; ^2^Department of Histopathology, Torbay Hospital, UK; ^3^Department of Otolaryngology, Torbay Hospital, UK

## Abstract

**Background:**

Inflammatory pseudotumours (IPT) are rare benign tumours characterised by spindle-shaped histiocyte proliferation often mimicking a soft tissue sarcoma. They can occur in different parts of the body and various aetiological factors have been proposed. To our knowledge this is the first case report of IPT of the larynx caused by mycobacterial disease.

**Case Report:**

We report a case of IPT of the larynx in an immunocompromised 81-year-old lady presenting with stridor and dysphagia with known disseminated* Mycobacterium kansasii* of the lungs.

**Conclusion:**

This case demonstrates both the clinical and histological difficulties in making the diagnosis of IPT. A high index of suspicion is needed, and the importance of a multidisciplinary approach in the work-up, diagnosis, and management is highlighted.

## 1. Introduction

Inflammatory pseudotumours (IPT) are extremely rare lesions characterised by spindle-shaped histiocyte proliferation, appearances that may be easily mistaken for a soft tissue sarcoma. IPT is of unknown aetiology with various potential stimuli being proposed, e.g., trauma, inflammation, smoking, and infection.

There are only a handful of case reports of IPT affecting the larynx, and most are of unknown aetiology [1–6]. To our knowledge this is the first report of a spindle cell pseudotumour in the larynx caused by* Mycobacterium kansasii *thus adding to the weight of evidence of an infective aetiology.

## 2. Search Strategy

OVID and MEDLINE were searched for terms “Laryngeal neoplasm AND inflammatory pseudotumour”, “Larynx AND inflammatory pseudotumour”. “Spindle cell neoplasm and Larynx” and references of papers identified using above search were also searched to find further case reports.

## 3. Case Report

### 3.1. Presentation

An 81-year-old lady presented to the ENT department with increasing dysphagia, shortness of breath, and stridor. Her past medical history included radiotherapy to the mediastinum for Hodgkin's lymphoma 14 years prior to presentation and radiation induced interstitial pulmonary fibrosis. She was on long-term Azathioprin and Prednisolone immunosuppressive therapy for this. One year prior to presentation she was treated for a fungating moderately differentiated squamous cell carcinoma of the tip of the nose with surgical excision. Histologically this measured 12mm x 11mm x 3mm with involvement of the deep margin, with no perineural or lymphovascular invasion. There was no lymph node metastasis, and the patient underwent 5 sessions of radiotherapy at 35Gray to treat the deep margin.

The patient was also investigated for symptoms of a lower respiratory tract infection 5 months prior to the latest admission with stridor. A sputum culture grew* Haemophilus influenzae* and* Mycobacterium kansasii* and the patient was advised 2 years of rifampicin, ethambutol, and clarithromycin by the respiratory team as per the British Thoracic Society recommendations [7]. Due to the duration of work-up of the diagnosis and need for 3 confirmatory sputum samples, the patient only had 6 weeks of triple therapy treatment prior to presenting with stridor.

Examination of the patient during the acute admission using nasoendoscopy revealed a left anterior vocal cord granulation and an exophytic pedunculated lesion from the right vocal cord causing ball-valving of the glottic inlet and diminished right vocal cord mobility. With the patient's history in mind, the initial working diagnosis was squamous cell carcinoma and a differential diagnosis of mycobacterial disease.

**Figure 1:**
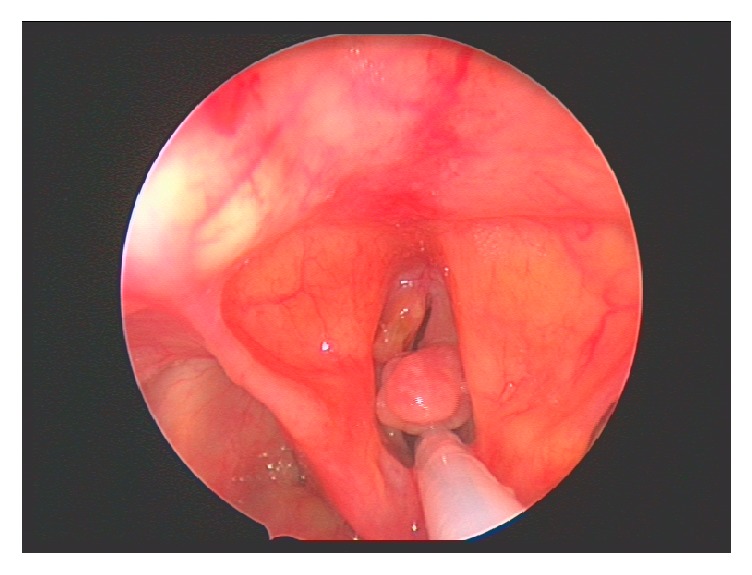
Direct laryngoscopy showing right vocal cord pedunculated lesion causing a bag-valve effect on the glottic inlet and left anterior vocal cord granulation.

**Figure 2:**
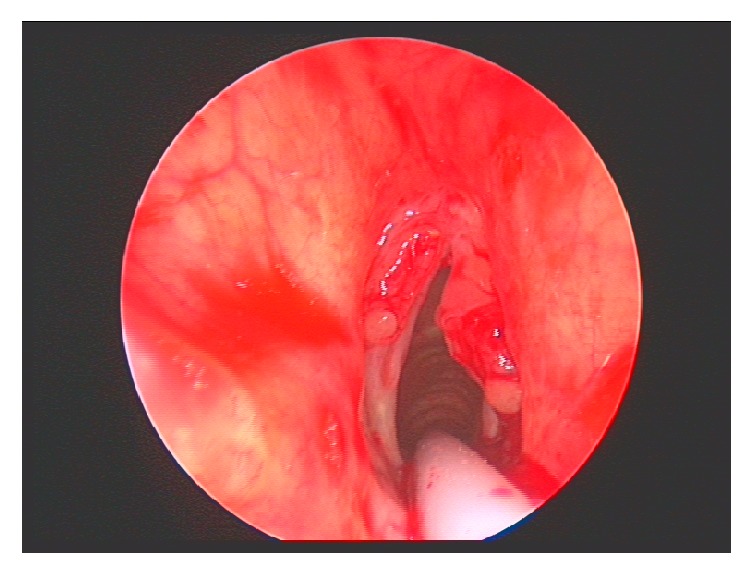
Direct laryngoscopy immediately following debridement of laryngeal lesions on both vocal cords to reestablish airway and to send tissue for histological and microbiological investigations.

The patient was initially commenced on broad-spectrum antibiotics and steroids. As there was no response to medical treatment, the patient was taken to theatre for debulking to reestablish an airway and tissue samples were sent for both histological and microbiological examination. Figures 1 and 2 reveal pictures of the larynx both before and immediately after debulking. The patient was discussed in the joint head and neck multidisciplinary meeting pending the results of histological analysis.

### 3.2. Histological Examination

Histology showed a spindle cell proliferation with intersecting fascicles and easily identifiable mitotic figures (Figure 4(a)). Initially the differential diagnosis included a low-grade sarcoma, especially as there was strong expression of desmin (Figure 4(b)) and weaker expression of smooth muscle actin. However, the lesion demonstrated bland uniform nuclei and there was an inflammatory cell infiltrate of neutrophils and plasma cells. Immunohistochemistry also showed expression of CD68, suggesting a significant histiocytic component to the lesion, but no true granulomata were identified. Focally the surface had a slightly palisaded appearance with necrotic debris on the surface, possibly representing an element of caseous necrosis. Staining with Ziehl-Neelsen (Figure 4(c)) and Wade-Fite (Figure 4(d)) showed numerous acid-fast bacilli.

The bland nuclear morphology, inflammatory cell infiltrate, and mycobacteria indicate an inflammatory pseudotumour secondary to mycobacteria infection. The lack of epithelioid granulomata may be related to the underlying immunosuppression. The strong expression of desmin and the weaker smooth muscle actin, although slightly unusual, would be acceptable for a myofibroblastic proliferation.

### 3.3. Following Management

**Figure 3:**
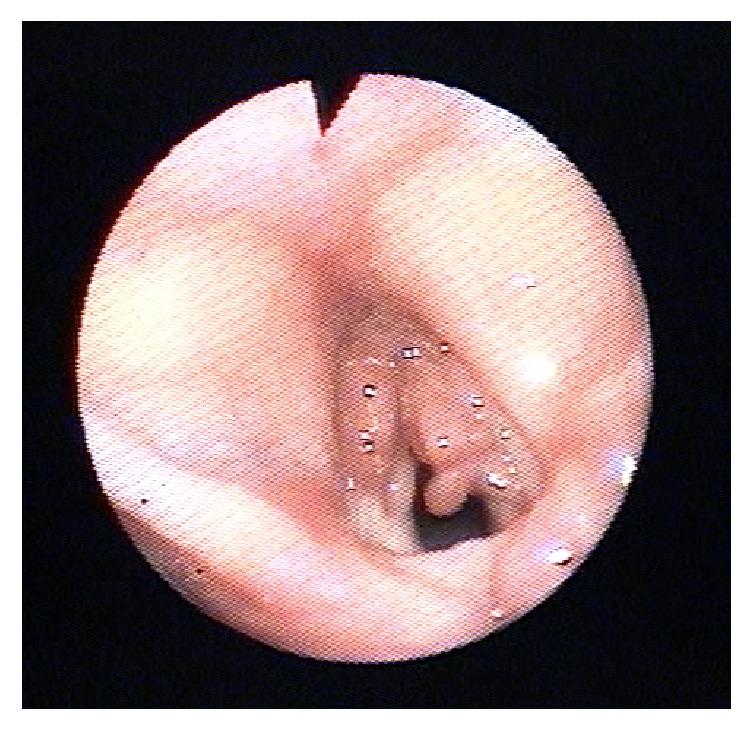
Indirect laryngoscopy on ward one week following debridement showing reproliferation of granulation tissue from both vocal cord and airway compromise.

**Figure 4:**
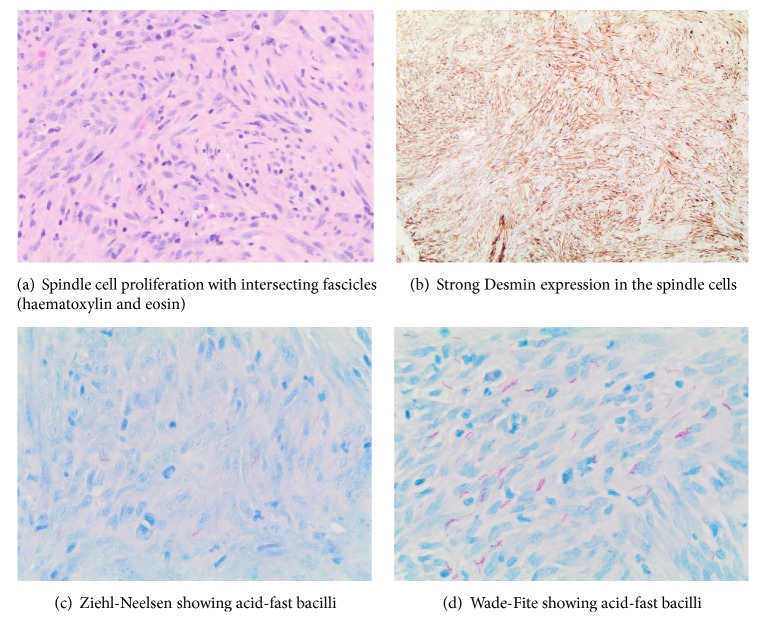


After confirmation of the histological diagnosis, the patient continued to show respiratory deterioration despite the surgical debulking and being started on broad-spectrum antibiotics and steroids. The long-term antimycobacterial medications were not stopped during the admission. Indirect laryngoscopy of the vocal cords was performed at the bedside one week after debulking showing reproliferation of the granulation tissue (Figure 3) with compromise of the airway. Given the overall poor prognosis and resistance to medical and surgical interventions, and taking the patient's wishes into consideration, treatment was withdrawn. The patient's condition unfortunately deteriorated following this and she died shortly afterwards.

## 4. Discussion

*Mycobacterium kansasii* is an acid-fast bacillus that causes pulmonary and extrapulmonary disease in immunocompromised patients, such as in HIV [8], type 2 diabetes [9], and organ-transplanted individuals [10]. It is also the commonest nontuberculous mycobacterium causing pulmonary infection in the UK [11]. IPT associated with mycobacterial disease has been reported in skin [12], lymph nodes [13], lung [14, 15], brain [16], and sinuses [17]. There have been cases of head and neck manifestations of IPT reported in lymph nodes, parotid, the para-pharyngeal space [18], and the tonsil [19]. IPT of the larynx are rare; in some cases there has been a history of intubation, previous surgery, or smoking [2]. To our knowledge this is the first case of* Mycobacterium kansasii* causing IPT in the larynx. This case adds weight to the theory of infective causation of pseudotumours of unknown aetiology.

### 4.1. Clinical Applicability

There are a few learning points from this case. Firstly, the diagnostic dilemma of a lesion in a patient with immunosuppression means that the initial working diagnosis should include infective causes and malignancy. For this reason, tissue should be sent for both histopathological and microbiological assessment. Although mycobacterial disease is rare in the UK, the rising numbers of patients on immunosuppressive therapy should alert the clinician to the possibility of rarer pathologies.

Secondly, this case illustrates the importance of a multidisciplinary approach when dealing with presentations of uncertain nature. The patient was discussed in a joint head and neck multidisciplinary meeting where opinions were exchanged between histopathologists and surgeons and the possibility of an inflammatory pseudotumour was raised. This suspicion was strengthened due to involvement of both vocal cords without continuity between lesions: it was felt that the occurrence of synchronous sarcoma in the larynx was highly unlikely.

### 4.2. Management

Once the diagnosis of mycobacterial inflammatory pseudotumour was confirmed, the management options were the subject of debate and a microbiologist was involved. Although the patient had maximal medical antimycobacterial therapy and surgical debridement, the pseudotumour recurred within one week. This was thought to be due to immunosuppression. Other case reports have advocated aggressive surgical excision in similar instances, although in this case that was not deemed appropriate.

Due to the rarity of laryngeal IPT, obtaining level-1 evidence to guide treatment is not practical, and case reports have generally guided treatment. In the more common setting of thoracic IPT of lungs and mediastinum complete surgical excision has been found to be effective [20]. Where surgery is not without considerable morbidity such as in orbital IPT, corticosteroids with or without radiotherapy have been found to show good disease control of 80%, however with high recurrence rates [18, 20]. Surgery with or without steroids is generally the preferred modality of treatment especially when there are easily accessible sites as the larynx [5]. Laryngeal lesions that are pedunculated and well defined can be surgically excised without need for adjuvant treatment. Adjuvant steroid therapy is advised for laryngeal lesions that are less well defined and affecting a large area of the glottis, supraglottis, or subglottis. Surgical debulking with a microdebrider reduces tumour bulk and reestablishes an airway but there is a high likelihood of leaving microscopic tumour behind. The duration of steroid treatment will depend on the extent of tumour involvement, patient comorbidities, and whether it is used as the sole modality of treatment or adjuvant therapy. Reported steroid regimes ranged from initial IV dexamethasone followed by oral corticosteroids for 2 weeks [5] in laryngeal IPT, to oral corticosteroids for a few months in orbital IPT with radiological monitoring of tumour response using MRI [20]. To monitor for recurrence, we propose that patients are seen regularly in the outpatients setting to have indirect laryngoscopy for at least 2 years after treatment is completed.

## 5. Conclusion

This case illustrates a rare presentation of mycobacterial inflammatory pseudotumour affecting the larynx. There are both clinical and histological difficulties in making this diagnosis as it may mimic a soft tissue sarcoma. A high index of suspicion for mycobacterial disease is needed in patients who are immunocompromised and the importance of a multidisciplinary approach in the work-up, diagnosis, and management of these rare conditions cannot be overemphasised.

## Additional Points

*Summary.* (i) Inflammatory pseudotumours are rare benign tumours characterised by spindle-shaped histiocyte proliferation and are of unknown aetiology. (ii) This is the first reported case of* Mycobacterium kansasii* causing inflammatory pseudotumour of larynx thus supporting an infective aetiology. (iii) A multidisciplinary approach is needed in the work-up, diagnosis, and management of such rare conditions.
